# Use of quantitative mass spectrometry-based proteomics and ELISA to compare the alpha 2 macroglobulin concentration in equine blood-based products processed by three different orthobiologic devices

**DOI:** 10.3389/fvets.2024.1335972

**Published:** 2024-02-09

**Authors:** Kyla F. Ortved, Larry Alward, Bobby Cowles, Renata Linardi, Dhvani Barot, Alex Usimaki, Joseph R. Fedie, Deb Amodie, Laurie R. Goodrich

**Affiliations:** ^1^New Bolton Center, University of Pennsylvania, Kennett, PA, United States; ^2^Veterinary Medicine Research and Development, Zoetis, Kalamazoo, MI, United States; ^3^Equine Technical Services, Zoetis, Parsippany, NJ, United States; ^4^Outcomes Research, Zoetis, Parsippany, NJ, United States; ^5^Orthopaedic Research Center, Translational Medicine Institute, College of Veterinary Medicine and Biomedical Science, Colorado State University, Ft Collins, CO, United States

**Keywords:** alpha 2 macroglobulin, equine, protease inhibitor, proteomic, regenerative joint disease therapy, selected reaction monitoring, orthobiologics

## Abstract

**Introduction:**

Alpha 2 macroglobulin (A2M), a multi-functional protein in the plasma protease inhibitor class, regulates proinflammatory cytokines and the clearance of chondrodestructive enzymes in cases of joint injury and osteoarthritis (OA). The purpose of this study was to compare A2M concentrations in equine plasma samples processed by three commercial devices developed for stall-side regenerative joint therapy.

**Methods:**

Plasma samples were obtained from healthy adult horses (*N* = 13). Mass spectrometry analysis was used to determine the concentration of protein analytes in each sample. Selected reaction monitoring measured a specific A2M peptide as a surrogate of the whole A2M protein. A2M concentrations produced by each test device were compared for two sample types: a pre-concentrate or platelet-poor (PP) component and a final component for use in the horse.

**Results:**

There was no significant difference (*p* > 0.05) in the geometric mean (GM) concentration of A2M in the final concentration samples produced by the Alpha2EQ^®^ device (N horses = 13) and the single-centrifugation PP samples produced by the Pro-Stride^®^ APS (autologous protein solution) device (*N* = 13) and the Restigen^®^ PRP (platelet-rich plasma) device (*N* = 11). When A2M content in final concentration samples produced by each device was compared, the Pro-Stride APS and Restigen PRP samples had significantly greater GM A2M content (*p* < 0.0001) compared to the Alpha2EQ samples, and the Pro-Stride APS final concentration samples had significantly greater GM A2M concentration (*p* < 0.0001) versus that for the Restigen PRP final samples.

**Discussion:**

This comparison demonstrated that the volume and A2M concentration of an Alpha2EQ final concentrate are no different than the volume and concentration of A2M in the PP from Pro-Stride or Restigen devices.

## Introduction

Alpha 2 macroglobulin (A2M) is a potent, naturally occurring protease inhibitor that is constitutively present in blood and at lesser levels in normal synovial fluid and cartilage of various vertebrate species, including horses (1, 2). A2M has inhibitory activity against all degradative endoprotease classes, particularly those activated by acute inflammation (2–6). This broad spectrum effect is a result of the A2M molecular structure, which has been described as a unique bait-and-trap mechanism (4, 7)

The antiprotease mode of action of A2M has been well characterized (1, 4–6, 8, 9). Briefly, the A2M molecule consists of a pair of covalently linked dimers forming a bait-region “cage” in the hollow core of the structure that is highly susceptible to cleavage by proteases (1, 6). When cleavage occurs, the A2M molecule undergoes an immediate conformational rearrangement that entraps the protease, resulting in inhibition of proteolytic activity and eventual clearance of the A2M-protease complex by the liver (4–6). In addition to protease neutralization, A2M binds to proinflammatory cytokines to reduce cytokine-induced synthesis of collagenases in cartilage (2, 3, 5, 8, 9). Thus, A2M has two principal cartilage-sparing effects: binding to proinflammatory cytokines that initiate the process of cartilage degradation and neutralizing catabolic enzymes that drive the progression of osteoarthritis (OA).

Since there is a rapid, multi-fold increase in its expression in response to inflammatory cytokines, A2M is considered to be an acute-phase protein (2, 10, 11) However, the intra-articular (IA) concentration of A2M in synovial fluid at the site of orthopedic injury is considerably less than that in serum, even in individuals with OA (2, 6) This is thought to be due to the large molecular weight of A2M, which impairs its ability to migrate from serum into synovial fluid (2, 12). Moreover, investigators have noted that because A2M inhibits proteases by conformational change of the A2M molecule followed by the elimination of the A2M-protease complex from circulation, neutralizing one or two protease molecules occurs at the expense of one molecule of A2M (6). This inherent insufficiency of A2M *in vivo* restricts its ability to fully inhibit the inflammatory cascade and the catabolic processes that cause OA (2, 12). Supplemental, autologous A2M administered by IA injection can overcome the limited availability of endogenous A2M.

Studies have demonstrated the ability of A2M to minimize chondrocyte death and cartilage matrix degradation in humans and various animal models (2, 9, 12). As such, A2M has been acknowledged as a viable clinical option for OA regenerative therapy, either as an individual treatment or as a component of multimodal co-therapy (2, 3, 6, 12, 13). This approach typically involves concentrating A2M and the removal of cellular components from the patient’s plasma by centrifugation or filtering for subsequent IA administration. The ability to administer autologous A2M as a point-of-care option promptly after processing it from whole blood is a considerable advantage given the rapid expression of chondrodestructive enzymes within hours following traumatic joint injury, Lieberthal et al. (14), Zhao et al. (15).

The purpose of this study was to compare A2M concentrations in equine plasma samples produced by three commercial devices (Alpha2EQ^®^, Pro-Stride^®^ APS, and Restigen^®^ PRP) developed for stall-side regenerative therapy. Mass spectrometry (MS), the gold standard for precise analysis of individual proteins in proteomic studies, Pusch et al. (16) was used to identify and quantify the A2M content in autologous samples. Two different MS experiments were performed in this study to measure the A2M content of the respective samples at recommended dosage sizes. First, a selected reaction monitoring (SRM) method was developed, as this is the preferred approach to measuring a protein from a very complex protein mixture such as plasma (17). Second, an untargeted proteomic profile method was developed to measure and compare the relative levels of the 16 most abundant plasma proteins for samples derived from two of the test devices. This is the first published report to quantify the A2M content in samples produced by any of the test devices.

## Materials and methods

### Test devices

Three commercial orthobiologic devices were used in the study: Alpha2EQ^®^ (Astaria, Houston, Texas, United States), Pro-Stride^®^ APS (Zoetis, Parsippany, New Jersey, United States), and Restigen^®^ PRP (Zoetis). Each device uses centrifugation to extract autologous regenerative and immunomodulatory biocomponents, including A2M, from a whole blood sample for subsequent IA injection into the host animal. Alpha2EQ uses two centrifugation cycles. The first device is to separate plasma from whole blood, and the second device is to isolate and filter A2M from plasma as a final concentrate. The Pro-Stride APS uses an initial centrifugation cycle to separate plasma, platelet-rich plasma, and red blood cells from whole blood. Platelet-poor (PP) plasma is withdrawn, leaving a cellular suspension of platelet-rich plasma, which is centrifuged in a second device with polyacrylamide beads to produce a final concentrate of autologous protein solution (APS) for therapeutic injection. The Restigen PRP uses a single-centrifugation cycle to separate and remove PP plasma, platelet-rich plasma (PRP), and red blood cells from the sample. The remaining PRP is used as the final concentrate for therapeutic injection.

### Horses

Healthy adult horses (*N* = 13) of various breeds (quarter horse and thoroughbred types) with ages ranging from 2 to 10 years and from geographically dispersed locations in the United States (a private practice in Missouri, University of Pennsylvania, and Colorado State University) were used as test animals. Horses were excluded if they were being treated with therapeutic medications for any preexisting condition. Horses at the Pennsylvania and Colorado locations were handled and maintained in compliance with the U.S. Department of Agriculture Animal Welfare Act (7 USC 2131) and the Institutional Animal Care and Use Committee (IACUC) guidelines in force at their respective institutions.

### Sample collection and processing

Whole blood samples were obtained using an 18-gauge needle with a short extension set and a 60-mL syringe from the jugular vein following sterile preparation. The syringes for each orthobiologic device contained 5 mL of anticoagulant citrate dextrose-A and 55 mL of whole blood. The samples were processed at the collection site by independent veterinarians not associated with the study design or interpretation of the data. The volume of plasma concentrate generated for MS and ELISA analysis was 30 mL. All samples were processed by the test devices within 1 h after collection, according to the manufacturers’ recommendations. Aliquots of each sample from 11 of the test horses were processed by all three test devices. Samples from two other horses were processed by the Alpha2EQ and Pro-Stride APS devices only. Table 1 summarizes the sample collection protocol and types of assays performed for each sample type.

**Table 1:** Sample collection and testing protocol.

Test device	Sample for analysis	Sample allocation and test
Alpha2EQ	First centrifugation: Pre-final plasma sample (*N* = 13)	3 mL for ELISA 3 mL for MS analysis of A2M content
	Second centrifugation: Final plasma sample (*N* = 13)	3 mL for ELISA 3 mL for MS analysis of A2M content and proteomic profile
Pro-Stride APS	First centrifugation: Platelet-poor (PP) plasma sample (*N* = 13)	2 mL for ELISA 4 mL for MS analysis of A2M content and proteomic profile
	Second centrifugation: Autologous protein solution (APS) sample (*N* = 13)	1.5 mL for ELISA 1.5 mL for MS analysis of A2M content
Restigen PRP	First centrifugation: Platelet-poor (PP) plasma sample (*N* = 11)	2 mL for ELISA 4 mL for MS analysis of A2M content
	First centrifugation: Platelet-rich plasma (PRP) sample (*N* = 11)	2 mL for ELISA 4 mL for MS analysis of A2M content

A2M, alpha 2 macroglobulin; ELISA, enzyme-linked immunosorbent assay; MS, mass spectrometry.

### Study design

The study compared the concentrations of A2M in equine blood samples processed by each of the three orthobiologic devices. A2M concentrations produced by each test device were compared for two sample types: a pre-concentrate or platelet-poor (PP) component and a final or cell-rich concentrate. SRM analysis was used to determine the A2M concentration in each sample. A2M concentrations were also measured by ELISA testing as a potentially rapid, high-specificity alternative to LC–MS analysis. Proteomic profiles were developed for a 7-horse contingent from the Colorado State University location and Missouri (UPENN samples for this process were excluded due to a sample storage issue). The proteomic profiles were determined by LC–MS analysis and consisted of the 16 most abundant proteins in the final samples produced by the Alpha2EQ device and in PP samples produced by the Pro-Stride APS device.

### Mass spectrometry: selected reaction monitoring

The SRM assay measured a specific A2M peptide as a surrogate of the whole A2M protein (Table 2). The tryptic peptide LLVYTILPDGEVVGDSAK was measured across sample groups in a targeted, SRM quantitative assay after trypsin digestion of all plasma proteins (trypsin cleaves protein after each arginine and lysine amino acid). The three types of peptide analytes in this assay were standard, target, and internal control. Standard and target peptides were identical but derived from synthetic and endogenous sources, respectively. The standard peptide was used to make a series of peptide concentrations to generate a standard curve. The target peptide was enzymatically liberated from the A2M protein in the test samples. The synthetic internal control peptide was identical to the corresponding target and standard peptide amino acid sequence except that the C-terminal lysine residue was substituted with a stable isotope-labeled form of lysine. While physiochemically identical, the stable isotope-labeled peptide is +8 mass units heavier than the target or standard peptides. The same amount of internal control peptide was spiked into every sample (including standard peptides) to account for variations due to matrix effects on ionization and instrument performance.

**Table 2:** Alpha 2 macroglobulin peptides analyzed with mass spectrometry.

Amino acid sequence	Peptide type	Molecular mass (Da)	Source
LLVYTILPDGEVVGDSAK	Standard	1889	Synthetic^b^
LLVYTILPDGEVVGDSAK	Target	1889	Equine plasma
LLVYTILPDGEVVGDSAK^a^	Internal control	1897	Synthetic^b^

a Denotes lysine residue that is stable-isotope labeled.

Da, Daltons.

b Custom orders purchased from Biosynth^®^(Gardner, MA, United States).

The plasma samples were trypsin-digested as follows: Each plasma sample was diluted 1:100 (20 μL of plasma into 1980 μL of 50 mM Tris pH 8.5). Two analysis volumes, 20 μL and 40 μL, were analyzed in duplicate in a 96-well plate. Next, 50 mM Tris, pH 8.5 was added so that each sample well contained 120 μL. Standard peptide solutions were added to the plate at 120 μL per well. Then, proteins in all wells were reduced for 60 min at 56°C in the presence of 5 mM Tris (2-carboxyethyl) phosphine, followed by alkylation for 30 min at room temperature in the presence of 6.3 mM iodoacetamide. Proteins were then trypsin digested (Promega Trypsin Gold, Mass Spectrometry Grade (#V5280), 100 mg/vial, stored at ≤ − 20°C) for 17 h at 37°C. The trypsin solution also contained an internal control peptide, so each well received consistent amounts of trypsin (3 μg) and internal control peptide (9 ng). Digestion was halted with the addition of formic acid (0.5% v/v).

SRM was conducted using a Waters TQS-Micro^®^ triple quadrupole mass spectrometer to measure A2M peptide concentration (nanomoles of A2M peptide/mL). Digested plasma peptides were analyzed using reversed-phase liquid chromatography (Waters CORTECS™ C18 2.7 μm column, 2.1 × 50 mm) that was eluted into the mass spectrometer. The first quadrupole of the mass spectrometer was used as a mass filter. Only analytes within one mass unit of the peptide masses in Table 2 were passed through to the second stage of the mass spectrometer. The second quadrupole of the mass spectrometer was programmed to fragment the peptides into a sequence-specific product ion by collision-induced dissociation. The third quadrupole increased the specificity by mass filtering the fragment ion pool to only allow the A2M sequence-specific fragment ion to pass through to the ion detector for quantitation. The mass spectrometer recorded peak area ion counts of the target peptides and the internal control peptide. The ratio of the target peptide ion count to the internal control peptide ion count was the response. A standard curve of the responses (*y*-axis) from known concentrations of standard peptide (*x*-axis) was plotted, and a linear 1/X-weighted fit was performed. The response from each unknown was interpolated from the standard curve to determine the A2M peptide concentration (nmol of A2M peptide/mL). Two input volumes from a sample were analyzed in duplicate, and the average was reported as the final A2M peptide concentration.

### Mass spectrometry: proteomics analysis

Trypsin-digested samples were spiked with a peptide mix standard (*E. coli* ClpB, Waters #186006012) and analyzed using a Waters Synapt^®^ G2Si quadrupole time-of-flight mass spectrometer. The peptide solution was analyzed using nanoscale reversed-phase liquid chromatography (Waters nanoEase™ M/Z HSS C18 T3 1.8 μm column, 75 μm x 200 mm) that was eluted into the mass spectrometer. Using the Waters UPLC-MS^E^ technology, the relative levels of the most abundant plasma proteins in each sample were measured. First, protein identifications were made by matching MS^E^ continuum data to an *Equus caballus* proteome database (with the peptide standard *E. coli* ClpB sequence added) using Waters ProteinLynx Global Server™ (PLGS) software version 3.0.3. Then, for each protein identified (including the spiked peptide standard), the software calculated the average MS signal response for the three most intense tryptic peptides. The average MS signal response from the spiked peptide standard was used to determine a universal signal response factor (counts/mol of protein), which was then applied to the other identified proteins in the mixture to determine their corresponding concentration. The quantity of each protein in the mixture was determined by dividing the average MS signal response of the three most intense tryptic peptides of each protein by the universal signal response factor described above (18).

### ELISA analysis for alpha 2 macroglobulin

All ELISA components, standards, and wash buffer were prepared on the day of analysis according to the manufacturer’s instructions (MyBioSource, San Diego, CA equine-specific A2M MBS014509). A 50 μL volume per well of standards and 50 μL per well of plasma were added to the appropriate wells of the pre-coated, commercially available plate. A 100 μL volume of anti-A2M antibody was added to the sample wells. After a 50 μL volume of streptavidin–HRP was added to each well, the plate was incubated for 1 h at 37°C. The plate was rinsed five times with 300 μL of wash buffer per well. A 50 μL volume each of substrate solution A and substrate solution B were added to each well, after which the plate was incubated for 10 min at 37° C. A 50 μL volume stop solution was added to each well. The plate was evaluated with a spectrophotometer at a wavelength of 450 nm against a 4-parameter logistic regression curve. Samples with a coefficient of variation >10% were re-evaluated, and if the standard curve for the plate had an *R*^2^ value <0.9, the entire plate was re-evaluated.

### Statistical analysis

The test animal was the experimental unit for the study. The primary variable of interest was the measurement of A2M. Measurements from all methods were checked for normality. A2M peptide was not normally distributed and, therefore, log transformed by the log (response) transformation prior to statistical analysis to stabilize the variance and normalize the data. All measurement data were analyzed using a generalized linear mixed model approach. Using the SAS Proc Mixed Procedure (SAS 9.4, Cary, North Carolina, United States), data were analyzed with a model that considered the fixed effect of the method and the random effects of the university and residual error. Least square means (LSMs) were calculated and compared by the two-sided Student’s t-test at a 5% level of significance. Transformed A2M peptide LSMs were back-transformed and reported as geometric means as well. No randomization was used for the study, and study administrators were not masked. The primary variable for the study was the quantitative measurement of A2M using the methods described.

## Results

### SRM measurement of A2M concentration in autologous plasma samples

**Figure 1:**
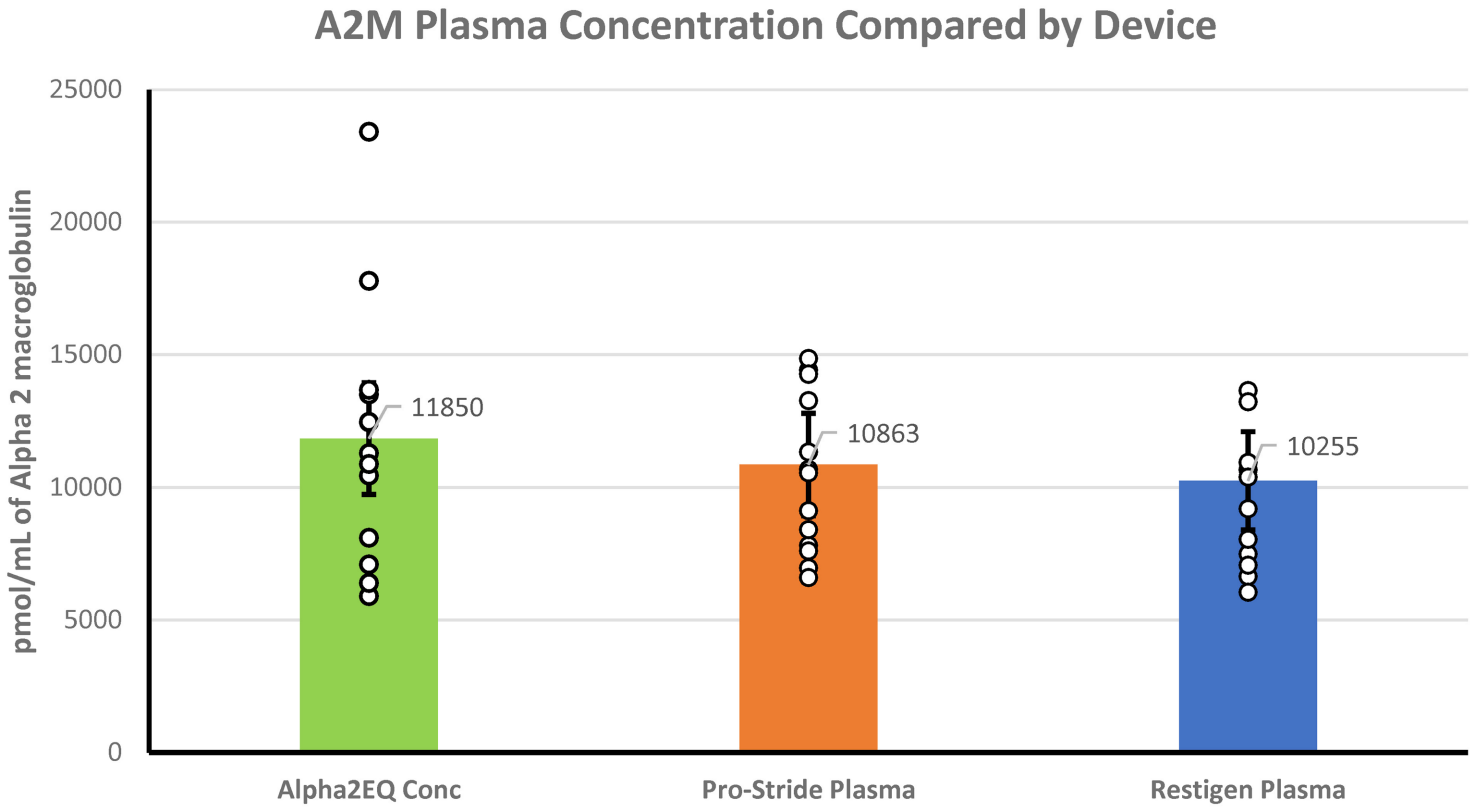
There was no significant difference (*p* > 0.05) in the geometric mean A2M concentrations in final equine plasma samples produced by Alpha2EQ (*N* = 13) versus pre-final, platelet-poor (PP) samples produced by Pro-Stride (*N* = 13) or Restigen (*N* = 11). The Alpha2EQ device used two centrifugation cycles to produce a final concentrate, which was compared to single-centrifugation, PP plasma samples produced by Pro-Stride or Restigen.

Mass spectrometry analysis determined that there were no significant differences (*p* > 0.05) in the geometric mean (GM) concentration of A2M in the Alpha2EQ final concentration samples (*N* horses = 13) and the single-centrifugation, as well as in the PP samples produced by Pro-Stride (*N* = 13) and Restigen (*N* = 11) (Figure 1). In this assay, where Alpha2EQ final concentrate was compared to PP samples produced by Pro-Stride and Restigen, the sample volumes and the amount of A2M delivered were equivalent.

**Figure 2:**
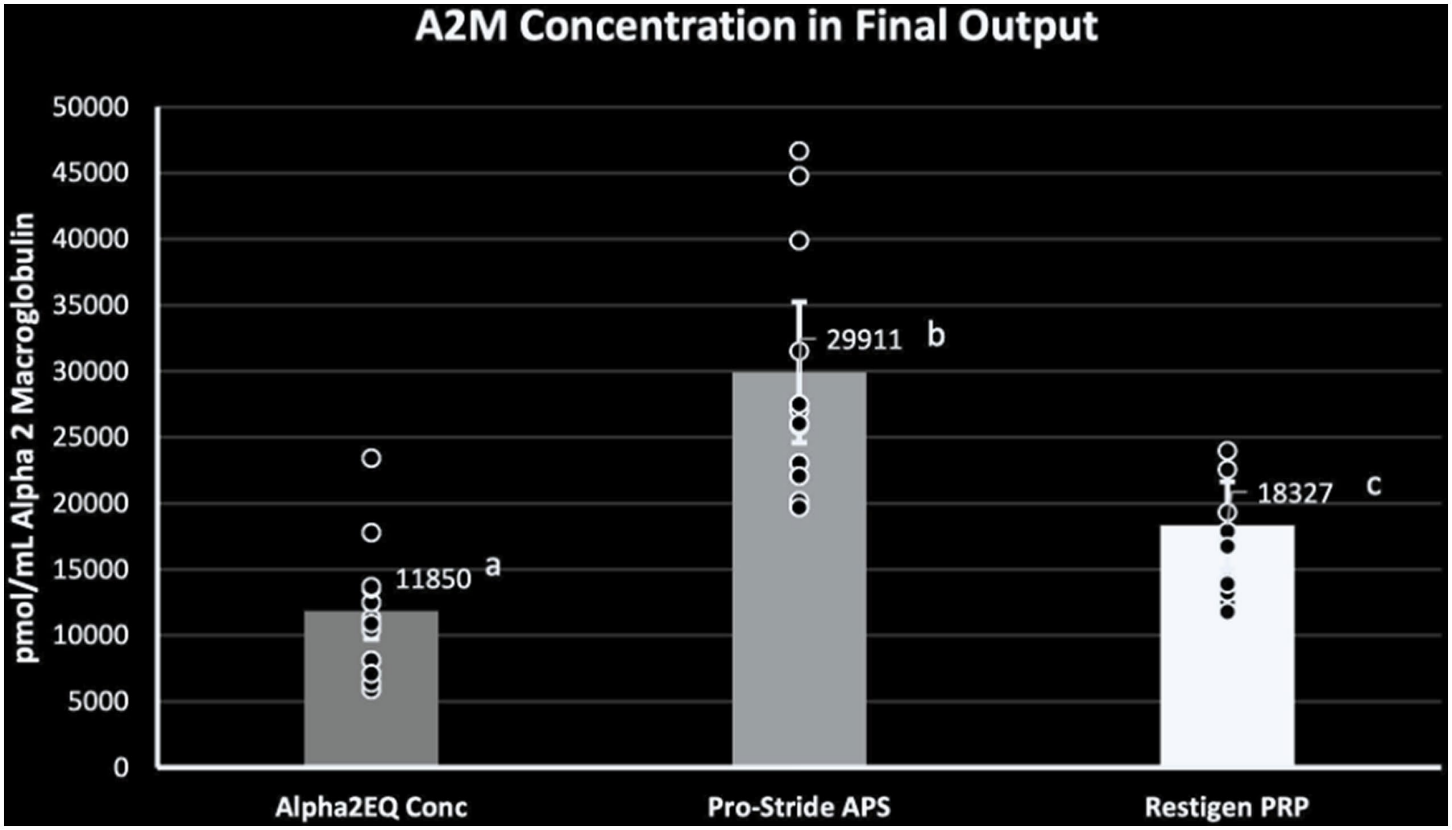
Geometric mean (GM) A2M concentration was significantly greater (*p* = 0.0001) in the final equine plasma samples produced by Pro-Stride APS (*N* = 13) or Restigen PRP (*N* = 11) compared to final samples produced by Alpha2EQ (*N* = 13). The GM A2M concentration in the Pro-Stride APS final samples was significantly greater (*p* = 0.0001) than the GM A2M concentration in Restigen PRP final samples. Columns with different superscripts indicate a significant difference between devices.

When the amounts of A2M in final concentration samples produced by each device were compared, the Pro-Stride APS and Restigen PRP samples had a significantly greater (*p* < 0.0001) GM A2M content compared to the Alpha2EQ final samples, and the Pro-Stride APS final samples had a significantly greater (*p* < 0.0001) GM A2M concentration versus that for the Restigen PRP final samples (Figure 2). This comparison demonstrated that the volume of an Alpha2EQ sample would have to be 2.5 times greater than a Pro-Stride APS sample to provide an equivalent amount of A2M.

**Figure 3:**
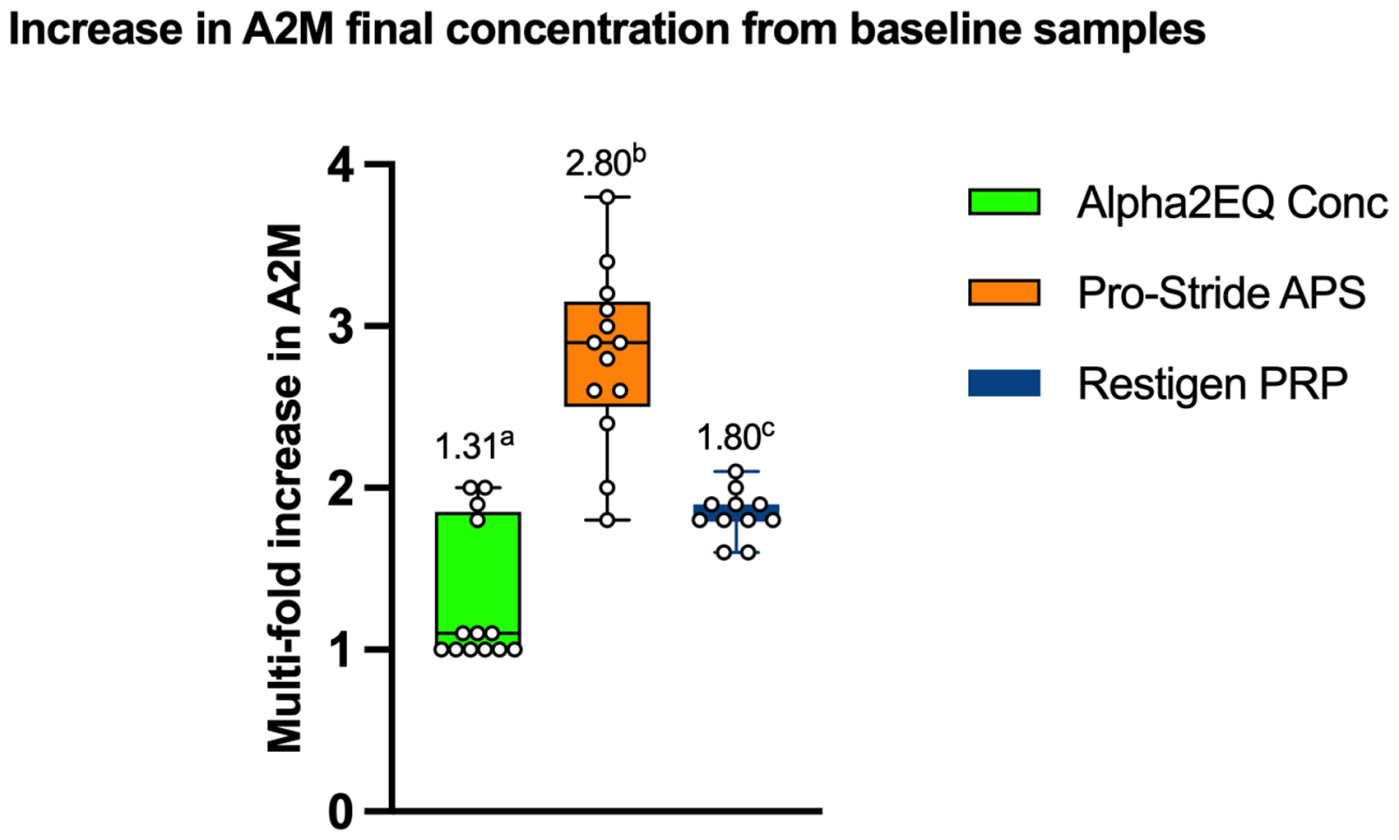
Increase in mean A2M concentrations from pre-final samples to final samples was significantly different (*p* < 0.05) among the three test devices. (Figures above each box Whisker Plot) Columns with different superscripts indicate a significant difference between test devices.

Figure 3 compares the mean increase in A2M content from the pre-concentrated plasma versus the final samples for each device that would be used therapeutically. The Pro-Stride APS final samples had a mean of 2.8 (median 2.85)-fold increase in mean A2M concentration versus the pre-final PP sample, which was significantly greater (*p* < 0.05) than the 1.8 (median 1.81)-fold and 1.31 (median 1.07)-fold mean increases, respectively, produced by the Restigen PRP and Alpha2EQ devices. Restigen PRP final samples had a significantly greater (*p* < 0.05) mean increase in GM A2M concentration compared to that for Alpha2EQ final samples (1.31 (median 1.07)-fold mean increase). Seven of the 13 Alpha2EQ samples had no A2M increase in the final concentration product. This contrasted with results for the Restigen PRP and Pro-Stride APS final samples, all of which had increased A2M concentrations compared to the pre-final samples.

**Figure 4:**
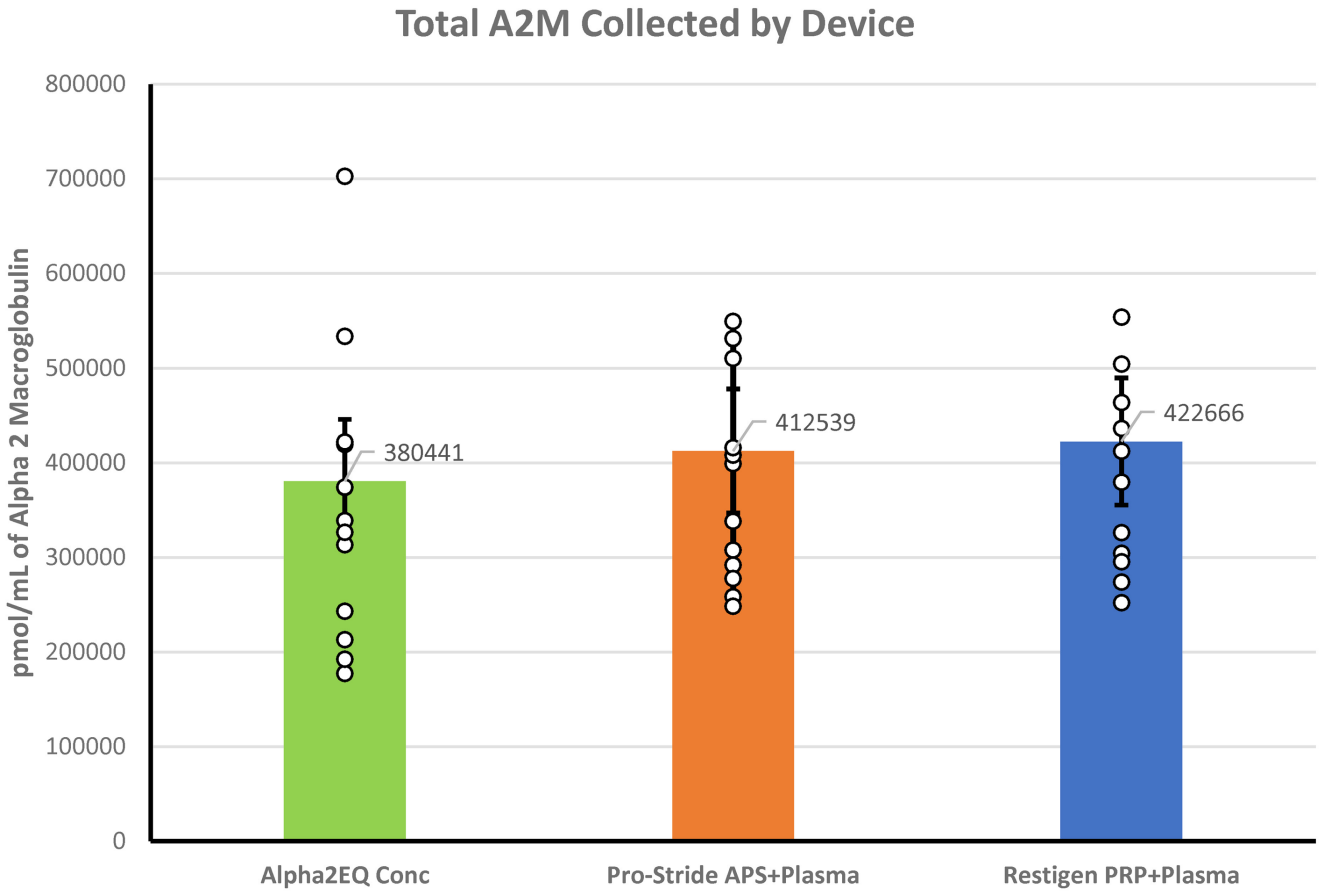
Aggregate amount of A2M measured in all samples collected for each of the three test devices was not significantly different (*p* > 0.30). The A2M total content in the Alpha2EQ final samples (*N* = 13) was measured as the geometric mean (GM) of the final concentration samples (*N* = 13). For the Pro-Stride APS and Restigen PRP samples (*N* = 13, 11, respectively), the total A2M content was calculated as the GM mean of the plasma poor (PP), pre-final sample plus the GM of the final sample.

There was no significant difference (*p* > 0.39) in the aggregate amount of A2M collected in all samples processed by each of the three test devices (Figure 4). The total GM A2M content was calculated for each test device as follows:


Alpha2EQ=GMA2Minthefinalsample×30mLofplasma.

$$\begin{array}{l} \mathrm{Pro}-\mathrm{stride}\;APS=GM\;A2M\;\mathrm{in}\;\mathrm{the}\;PP\;\mathrm{sample}\times 30\;\mathrm{mL}+\\ \;GM\;\mathrm{in}\;\mathrm{the}\;\mathrm{final}\;APS\;\mathrm{sample}\times 2.5\;\mathrm{mL}\text{.} \end{array}$$

$$\begin{array}{l} \mathrm{Restigen}\;PRP=GM\;A2M\;\mathrm{in}\;\mathrm{the}\;PP\;\mathrm{sample}\times 30\;\mathrm{mL}+\\ \;GM\;\mathrm{in}\;\mathrm{the}\;\mathrm{final}\;PRP\;\mathrm{sample}\times 6\;\mathrm{mL}\text{.} \end{array}$$

### ELISA measurement of A2M concentration In autologous plasma samples

**Figure 5:**
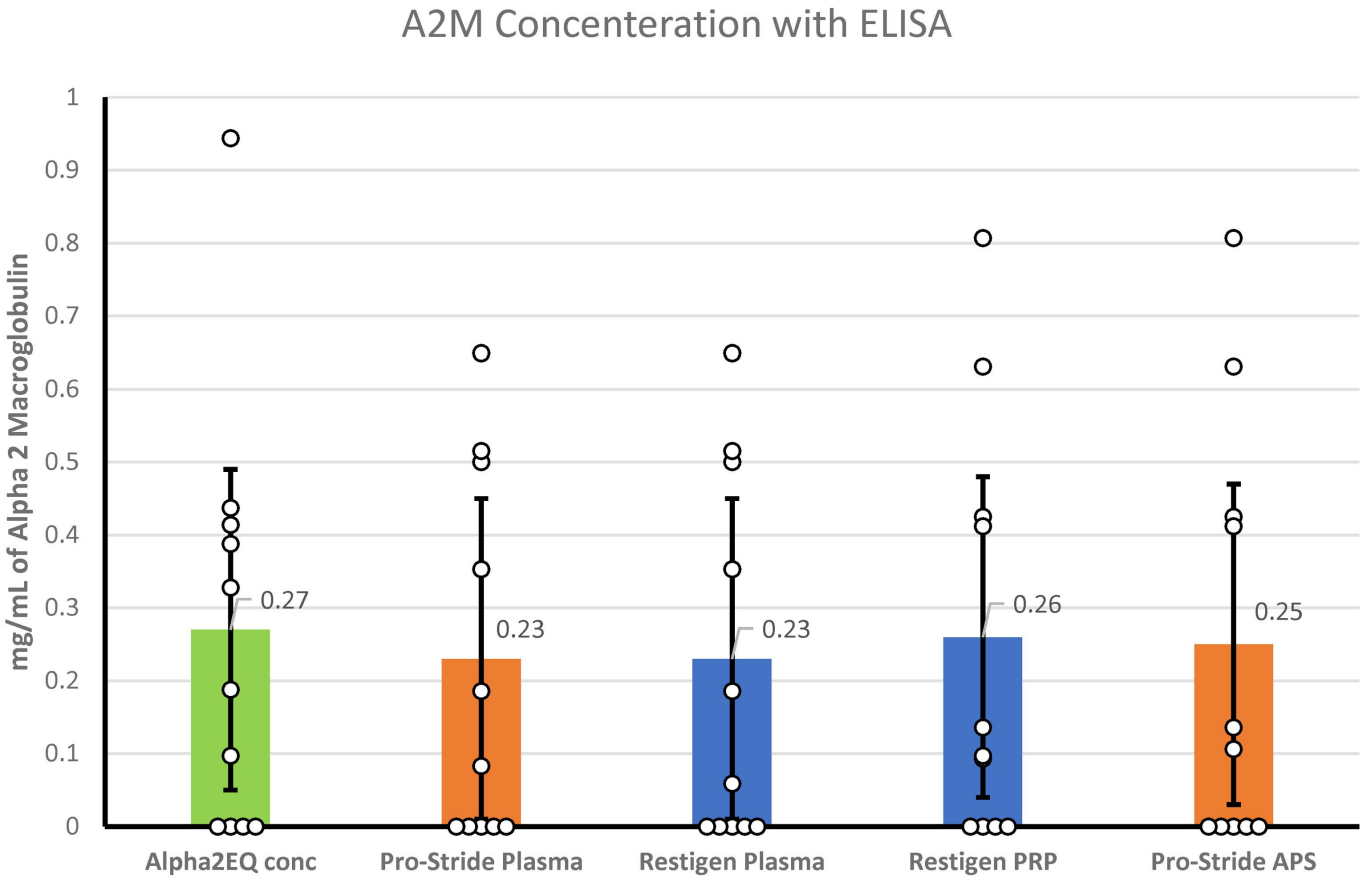
When measured by ELISA, there was no significant difference (*p* > 0.3) in the least squares mean concentration of A2M in Alpha2EQ final concentrate samples (*N* = 13), Pro-Stride platelet-poor (PP) plasma (*N* = 13), Pro-Stride APS platelet-rich plasma (PRP, *N =* 13), Restigen PP (*N* = 11), or Restigen PRP plasma (*N* = 11) samples.

ELISA testing for the A2M concentration was performed for the following samples: Alpha2EQ final concentration samples, Pro-Stride PP and final concentration (APS) samples, and Restigen PP and final concentration (PRP) samples. LS mean values were used for analysis as there was minimal variation in A2M content among the samples (Figure 5). Although Alpha2EQ concentrate had a small numerical advantage in LS mean A2M content versus the other samples, there was no statistical difference (*p* > 0.3) in the LS mean A2M concentration in the five types of plasma samples.

### Proteomics analysis of abundant proteins in plasma samples

Proteomic profiles of the 16 most abundant plasma proteins in the Alpha2EQ final concentrate and Pro-Stride PP samples were expressed as mg/30 mL concentrations (Table 3). There was little individual variation in A2M levels among the 7-horse test group or between mean A2M levels in samples produced by the two test devices (57.9 and 56.3 mg/30 mL, respectively, for Pro-Stride PP and Alpha2EQ samples). Concentrations of the other plasma proteins were also comparable between samples from the two test devices, except for immunoglobulin heavy constant mu (IGHM). IGHM levels varied >10-fold among individual horses, and mean IGHM values were substantially different between test devices (61.6 and 37.8 mg/30 mL, respectively, for Pro-Stride PP and Alpha2EQ samples) but not statistically significant (*p* > 0.3).

**Table 3:** Proteomic profile for the 16 most prevalent proteins in Pro-Stride platelet-poor (PP) plasma and Alpha2EQ final concentrate.

Plasma protein	Mean concentration and range (mg/30 mL)	
	Pro-Stride PP	Alpha2EQ
Albumin	542.8 (345.6–731.7)	579.0 (260.1–775.7)
Alpha 2 macroglobulin	57.9 (39.9–78.9)	56.3 (33.7–72.7)
Fibrinogen alpha chain	41.9 (21.0–60.3)	37.5 (15.2–62.1)
Serotransferrin	24.6 (18.3–33.7)	25.9 (18.9–38.3)
Fibrinogen gamma chain	14.2 (6.6–24.6)	15.0 (6.7–25.9)
Fibrinogen beta chain	14.0 (5.8–22.8)	12.7 (5.4–19.3)
Apolipoprotein E	14.8 (10.8–19.8)	15.1 (9.3–18.7)
Immunoglobulin heavy constant mu	61.6 (5.5–153.9)	37.8 (5.1–121.8)
Ig-like domain-containing protein	10.8 (2.7–23.0)	12.1 (2.9–24.5)
Alpha 2-HS-glycoprotein	6.8 (4.5–9.0)	7.1 (4.8–8.6)
Fibrinogen alpha chain (fragment)	0.56 (0.2–0.9)	0.5 (0.1–0.9)
Haptoglobin	11.8 (3.6–30.0)	12.7 (3.2–34.1)
Histidine rich glycoprotein	5.4 (2.7–9.8)	6.6 (2.5–8.9)
Antithrombin-III	1.9 (1.1–2.6)	2.0 (1.1–2.6)
Hemopexin	2.1 (0.7–3.3)	2.3 (0.6–4.1)
Apolipoprotein A-II	1.3 (0.0–2.5)	1.5 (0.6–2.5)

## Discussion

Although conventional equine parenteral joint therapies such as IA corticosteroids and IA hyaluronic acid can provide symptomatic relief in cases of OA, regenerative or cell-based therapies such as A2M, APS, and PRP have potential disease-modifying as well as palliative effects. This distinction makes autologous blood-derived products and cell-based therapies a promising component of comprehensive treatment for osteoarthritis. Orthobiologics that deliver A2M, APS, and PRP provide an additional therapeutic option for treating equine joint injury, potentially reducing reliance on IA corticosteroids, the current mainstay for anti-inflammatory OA treatment. No sector of veterinary medicine would benefit from this therapeutic approach more than equine practice, where an estimated 80% of horses >15 years and a third of Thoroughbred racehorses as young as 2–3 years of age are affected by well-established OA lesions (19–21).

The chondroprotective effect of Pro-Stride APS on equine cartilage and its clinical efficacy have been established in prior studies, Bogers (19), Bertone et al. (22), Linardi et al. (23), Velloso Alvarez et al. (24) and the positive effects of PRP in equine OA and tendon repair have been described (19, 25–27). However, Pro-Stride APS and Restigen PRP are not specifically branded for providing autologous A2M. In addition to the APS and PRP components of blood samples produced by these devices, our study demonstrated that Pro-Stride APS and Restigen PRP also provide A2M in per-dose quantities significantly greater than those derived from Alpha2EQ, a device labeled specifically for delivering A2M for use as equine joint therapy (Figure 2). Study results found that equine blood samples produced by Pro-Stride PP or Restigen PP contained amounts of A2M equivalent to final concentrate samples produced by Alpha2EQ (Figure 1). Final concentrate solutions of Pro-Stride APS and Restigen PRP contained significantly greater amounts of A2M/mL than Alpha2EQ on a per-dose basis (Figure 2) and contained significantly more A2M than PP samples (Figure 3). The implication is that relatively smaller doses of Pro-Stride APS and Restigen PRP concentrate can be expected to deliver an equivalent therapeutic effect as a larger dose volume of Alpha2EQ concentrate.

When used according to label directions, Pro-Stride APS or Restigen PRP produces a 30-mL volume of a PP solution, in effect a reservoir of A2M-containing plasma in addition to the final concentrate product. Practitioners could choose to use the residual PP concentrate to supplement multiple doses of the final concentrate to deliver a therapeutic dose of A2M in addition to APS (given at approximately 3 mL per dose) and PRP (given at approximately 6 mL per dose). Stated another way, Pro-Stride APS and Restigen PRP yielded two products for use in equine joint therapy, while Alpha2EQ yielded one product. Pro-Stride APS produces 2.5 mL of a comprehensive concentrate with plasma and cellular components, including platelets and WBCs that produce anti-inflammatory cytokines, plus 30 mL of PP plasma with significantly concentrated A2M and other plasma proteins for use in other joints. Restigen PRP produces 6 mL of platelet-rich plasma in addition to 30 mL of PP plasma with significantly concentrated A2M. Alpha2EQ yields a final approximate output of 30 mL of plasma with A2M and other plasma proteins but without all the cellular components available from the other two devices.

In contrast to SRM analysis, ELISA results showed no significant variations in A2M concentration and negligible amounts of A2M (<0.3 mg/dL) among the various test samples, suggesting that the ELISA used may not contain an antibody that binds equine A2M effectively. Because some samples were below the level of ELISA detection, differences in methodology or test materials (use of ACD-A as an anticoagulant versus EDTA or heparin) between laboratories may have also been involved.

There is considerable variation in the biological activity of equine cell- and protein-based products (19). This study establishes the capacity of the three study devices to deliver enhanced quantities of A2M well beyond the relatively low endogenous levels present in the equine host. The confirmed presence of concentrated quantities of A2M in the pre-final and final concentrate solutions produced by the Pro-Stride APS and Restigen PRP devices indicates that they deliver multiple regenerative agents. A solution that includes A2M as well as PRP or APS could be expected to provide clinically relevant anti-inflammatory, antiprotease, and regenerative effects in equine OA patients. Practitioners using these devices have the advantage of an autologous therapeutic option that reliably avoids adverse reactions and can be produced rapidly as a stall-side treatment, either as a stand-alone therapy or in a multimodal regimen.

## Data availability statement

The raw data supporting the conclusions of this article will be made available by the authors, without undue reservation.

## Ethics statement

The animal study was approved by Institutional Animal Care and Use Committee - University of Pennsylvania and Colorado State University. The study was conducted in accordance with the local legislation and institutional requirements.

## Author contributions

KFO: Conceptualization, Investigation, Methodology, Validation, Visualization, Writing – original draft, Writing – review & editing. LA: Conceptualization, Investigation, Methodology, Validation, Visualization, Writing – original draft, Writing – review & editing. BC: Conceptualization, Funding acquisition, Methodology, Validation, Visualization, Writing – original draft, Writing – review & editing. RL: Writing – review & editing. DB: Investigation. AU: Investigation. JRF: Investigation. DA: Data curation, Formal Analysis, Writing – original draft, Writing – review & editing. LG: Conceptualization, Investigation, Methodology, Validation, Writing – original draft, Writing – review & editing.
